# Importance of the Functions of the Skin in the Pathology and Treatment of Tubercular Consumption

**Published:** 1860-08

**Authors:** A. Toulmin

**Affiliations:** St. Leonard’s


*On the Importance of the Functions of the Skin, in the Pathology and Treatment of Tubercular Consumption.* By A. Toulmin, Esq., (St. Leonard's.)

The author commenced by offering as the proximate cause of tubercle in all cases, the breathing of impure air, and air in so small a quantity as to render it impure, especially during the night. Wherever this was the continuous state of existence, the result must be a deficiency of oxygen in the red corpuscles of the blood, and as the consequence of this, the deposition of plastic fibrine in an incomplete state of oxygenation, and therefore of organization, and thus incapable of being ultimately got rid of by change of matter. It consequently remained as an extraneous adventitious substance in the system offering to the observer all the characteristics of tubercle.

To explain the discrepancy which appears in the rich (who have no want of oxygen in the air they breathe,) being equally subject to phthisis with the poor, he drew attention to the importance of the respiratory functions of the skin, as proved by the almost instant death that occurs on closing the cutaneous pores by artificial means, as by varnishing and gilding the skin of rabits and other animals; and he observed that, in consequence of the coldness of our climate and other causes, the better classes of society were certainly not in the habit of making the washing the whole surface of the body a part of their daily toilet; and consequently that the exuviæ momentarily forming on the surface of the skin—the joint production of the sordes from within, combined with the *debris* of the cuticle—soon became more or less impervious, although the individual might be in the habit of changing his linen daily.

As an illustration of this state of skin, the author referred to acne so frequently seen on the face, as being in reality the general state of the skin of a large proportion of society especially in the earlier periods of life, when phthisis generally shows itself. The free entrance of air, as well as the exit of carbonic acid through the skin, being thus impeded, the same imperfect oxygenation of the blood, ensued as was produced in the poorer classes, by breathing mephitic air. For the removal of this state of the skin, the only means of cure were to be found in the instituting a full and diaphoresis by the aid of artificial heat; the result of which in first softening and then expelling large quantities of inspissated sebaceous matter, after the surface of the body had been washed clean with soap and water, was not surprising.

The use of hot air bath, as a therapeutic agent was no innovation on the established practice of the profession, as it was the mode of bathing practiced by Hippocrates, Galen and Celsus; and the universality of the practice was shown by the fact that the remains of such baths had been found in every colony of the Roman empire.

If tubercle be imperfectly organized fibrine, then it should be looked upon as a blood disease; and, seeing it is found in other parts besides the lungs, without destroying life, its deposition in them should not be considered as disease either of the lungs or air-tubes, but as an accidental circumstance, killing mechanically, by its ulcerations extending to the surrounding lung tissue. The author called in question the propriety of sending consumptive patients abroad to a warm climate during any stage of the disease; as although in the latter stages of the complaint, when the air tubes sympathized with the tubercular irritation, a warm atmosphere seemed more congenial to the patient's feelings; still in the earlier stages, when a cure was practicable, the breathing the open air of our winter, (at least on the south side of the island,) was most important. He instanced, as proof that the breathing cold air did not cause the complaint, the fact that tubercular consumption is not to be met with in high northern latitudes.

The treatment of phthisis was considered under its hygienic and medical aspects. Under the former, and particularly in the earlier stages, the patient was recommended to live in a high, dry and marine atmosphere, on the Downs, rather than under them; to be as much as possible in the open air; to use all sorts of athletic exercises, (avoiding such as accelerate the pulmonic circulation) suitable to the strength and sex of the patients, by which a more rapid change of matter is effected, together with absorption of already deposited tubercle; as well as the deposition of more healthy—*i. e.*, of more highly organized matter. Medically, the treatment was comprised in a few short aphorisms, which were: 1. The keeping the functions of the skin in healthy action by means of the hot air bath. 2. The annointing the whole surface of the skin daily with some oleaginous matter. 3. The keeping a local ulceration always patent by means of an issue or seton ; and 4. The use of some one or more of a large variety of tonic and antiseptic medicines; all admirable adjuvants in improving the general health, (if selected in conformity with the function most sympathising with and reacting on the disease,) but powerless in arresting the specific lesion in question, without the previous "Open Sesame," of the hot air bath, followed by aspersion of cold or tepid water.—*Brit. Med. Journal.*

